# A Case of Irregularity

**Published:** 1893-06

**Authors:** P. J. Friedrichs

**Affiliations:** New Orleans.


ARTICLE II.

### A CASE OF IRREGULARITY.

BY P. J. FRIEDRICHS, D. D. S., NEW ORLEANS.

Read before the Louisiana State Dental Society, February, '93.

I regret that I cannot do more for this meeting of our Society than giving the report of an interesting case relating to the subject.

The correction of irregularities, or mal-position of teeth in the Dental arches, has received the attention of the best minds in the profession, and its difficulties, and complications have tested to the utmost, the skill and resources of the operator.

Much patience and earnest labor coupled with well directed efforts are necessary to accomplish successful results. However, there is no operation in our specialty which affords more satisfaction to both patient and operator than this one, besides it is always remunerative—clients are generally willing to pay handsomely for perfect results, and I may also add that the fee charged, whatever it may be, is well earned.

The subject of these remarks, a little girl, was 13 years of age when I saw her. The superior centrals protruded about three-eights of an inch beyond the corresponding lower teeth. The arches were somewhat contracted. The mouth on being closed did not entirely shut out from view the superior centrals, marring the pleasant expression of the face; aside from this peculiarity, it was intelligent and rather prepossessing, and of course, the parents were more than anxious that this deformity should be remedied.

After I had taken impressions of the case, and studied its peculiarities, I found it absolutely necessary to remove the first Bicuspids on either side, in order to get sufficient space to reduce the arch.

It took the little patient just two weeks to make up her mind to undergo the operation of extraction.

September 14, 1891, the plate for reducing the irregularity was adjusted. It was of the ordinary pattern, made of rubber, covering the palate and extending as far back, and including the first molars. The band also of rubber, passed around the arch on labial and buccal surfaces of the teeth. The band is firmly attached and secured to the palatal portion of the plate by means of two gold bands passing between Molars and Bicuspids, besides allowing the rubber to make connections through the space made by the extraction of the Bicuspids. This regulating appliance was made to fit very tight against the six anterior teeth. After it had been worn four days it became loose and a further tightening had to be made, but as there would have been too great a strain on the Molars and Bicuspids, and one which would have probably moved them forward, I resorted to separating rubber in order to relieve tention and get space. A piece of rubber was placed between Cuspid, Lateral and Central on either side, thus room was gained so that I could once more apply the band force for general reduction. In a few days the rubbers were removed, and the band tightened to force the Centrals back. The band was tightened by simply heating it over a spirit lamp, and with the fingers pressing it slightly inward and immediately cooling off in water. This process was continued until it became necessary to shorten the band. This was done by cutting a piece out of its center, and by heat and pressure bringing the two ends together.

In the meantime it became necessary to cut away the palatal portion of the plate immediately posterior to the receding teeth in order to relieve pressure caused by a cushioning of the soft tissues.

In the course of about two months the reduction of the irregularity was complete. The case was now ready for the retaining plate, which was made in every way similar to the one for correction, only it was less bulky in construction. This plate was worn constantly for about six months, after which time she only wore it at night. The importance of continuously wearing the retaining plate cannot be too strongly impressed upon the mind of the patient who had just undergone the pain and inconveniences attending the regulating process. It depends upon the faithful wearing of this plate if any permanent benefit and success is to be realized. The principles involved in the reduction of irregularities may be reduced to this simple statement:

1st. An appliance to meet the requirements of each individual case.

2. Its attachment in such a way that the necessary force can be applied to the teeth to be moved.

3. An appliance which will keep the teeth in situ until there is no further tendency to change position.

There is one other important thing to consider, and that is the age of the patient. In my opinion, no general regulating should be undertaken, until the cuspids have made considerable progress in eruption. By this time the changes which still take place are reduced to a minimum, and can therefore interfere but little, or retard the work to be accomplished.—*Southern Dental Journal.*

